# Supplementary material for: Fetal Tissue-Derived Mast Cells (MC) as Experimental Surrogate for In Vivo Connective Tissue MC
Source: Cells. 2022 Mar 8;11(6):928. doi: 10.3390/cells11060928 (PMC8946182; doi:10.3390/cells11060928)
Supplement: Supplementary file 1 [file cells-11-00928-s001.zip › Supplementary Table S1.pdf]

Primer	Forward primer (Sequence 5’-3’)	Reverse Primer (Sequence 5’-3’)
Mcpt1	GGAAAACTGGAGAGAAAGAACCTAC	GACAGCTGGGACAGAATGGGG
Mcpt2	ATTTCATTGCCTAGTTCCTCTGAC	AGGATGAGAACAGGCTGGGAT
Mcpt4	GACAGAATCCACACAGCAGAAG	CCTCCAGAGTCTCCCTTGTATG
Mcpt5	GGCAGAACAAACGTGAATGAGCC	AAGAACCTTCTGGAAGCTCAGGG
Mcpt6	GCTCCTCTCTTTGAACCGGATC	GGTGGGAGAGGCTCGTCATTA
Mcpt7	GGCTGGGGTAACATCGACAAT	CAAGTAATAGGTGACCCGGGTGTA
Mcpt9	TTCCAAGTTCAATGACATCGTATTAC	GATACTTTTTTCACTCCAGTTCGC
Cpa3	ACACAGGATCGAATGTGGAG	TAATGCAGGACTTCATGAGC
TLR1	ACTATGCTGGTGCTGGCTGT	CCAGGGCAGGTCAAAGTAGA
TLR2	GGGGCTTCACTTCTCTGCTT	AGCATCCTCTGAGATTTGACG
TLR3	GCTCAGAAAGGCCTGGAAAT	GCCCTTAAAAGCAACAACACTCTG
TLR4	GGACTCTGATCATGGCACTG	CTGATCCATGCATTGGTAGGT
TLR5	GCTACCCAGGTGGCAAGAG	GCACAGAAAGCATGAAGCTG
TLR6	CTCACCAGAGGTCCAACCTT	CGAGCACTTCCAGGTTGTTT
TLR7	AAACTCAGCTGTGACAGAATGG	TCTTGGATCTTCCAATTTTGC
TLR9	GAATCCTCCATCTCCCAACA	CCAGAGTCTCAGCCAGCACT
Actin	CTAAGGCCAACCGTGAAAAG	ACCAGAGGCATACAGGGACA
